# Etiology and Recovery of Neuromuscular Function Following Academy Soccer Training

**DOI:** 10.3389/fphys.2022.911009

**Published:** 2022-06-13

**Authors:** Ciaran Deely, Jamie Tallent, Ross Bennett, Alex Woodhead, Stuart Goodall, Kevin Thomas, Glyn Howatson

**Affiliations:** ^1^ Department of Sport, Exercise and Rehabilitation, Northumbria University, Newcastle-upon-Tyne, United Kingdom; ^2^ Queen Park Rangers Football Club, Crane Lodge Road, London, United Kingdom; ^3^ School of Sport, Rehabilitation, and Exercise Sciences, University of Essex, Colchester, United Kingdom; ^4^ Department of Physiotherapy, School of Primary and Allied Health Care, Faculty of Medicine, Nursing and Health Science, Monash University, Melbourne, VIC, Australia; ^5^ Centre for Applied Performance Sciences, Faculty of Sport, Allied Health and Performance Sciences, St. Mary’s University, Twickenham, United Kingdom; ^6^ Water Research Group, Faculty of Natural and Agricultural Sciences, North West University, Potchefstroom, South Africa

**Keywords:** neuromuscular function, voluntary activation, contractile function, CNS, peripheral mechanisms, soccer, recovery

## Abstract

**Aim:** To profile the etiology and recovery time-course of neuromuscular function in response to a mixed-content, standard training week in professional academy soccer players. We concurrently examined physical performance, cognitive function, and perceptual measures of mood and wellness states to identify a range of simple tests applied practitioners could use in the field as surrogate measures of neuromuscular function.

**Methods:** Sixteen professional academy soccer players completed a range of neuromuscular, physical, perceptual, mood, and cognitive function tests at baseline and after a strenuous training day (pitch and gym), with retest at 24, 48, and 72 h, and further pitch and gym sessions after 48 h post-baseline. Maximal voluntary contraction force (MVC) and twitch responses to electrical stimulation (femoral nerve) during isometric knee-extensor contractions and at rest were measured to assess central nervous system (voluntary activation, VA) and muscle contractile (potentiated twitch force, Q_tw,pot_) function.

**Results:** Strenuous training elicited decrements in MVC force post-session (−11%, *p* = 0.001) that remained unresolved at 72 h (−6%, *p* = 0.03). Voluntary activation (motor nerve stimulation) was reduced immediately post-training only (−4%, *p* = 0.03). No change in muscle contractile function (Q_tw,pot_) was observed post-training, though was reduced at 24 h (−13%, *p* = 0.01), and had not fully recovered 72 h after (−9%, *p* = 0.03). Perceptions of wellness were impaired post-training, and recovered by 24 h (sleepiness, energy) and 48 h (fatigue, muscle soreness, readiness to train). Countermovement jump performance declined at 24 h, while RSI (Reactive Strength Index) decrements persisted at 48 h. No changes were evident in adductor squeeze, mood, or cognitive function.

**Conclusion:** Elite youth soccer training elicits substantial decrements in neuromuscular function, which are still present 72 h post-strenuous exercise. Though central processes contribute to post-exercise neuromuscular alterations, the magnitude and prolonged presence of impairments in contractile function indicates it is the restitution of muscular function (peripheral mechanisms) that explains recovery from strenuous training in academy soccer players.

## Introduction

Association football (soccer) is characterised by intermittent bouts of high-intensity activity including accelerations, sprints, changes of direction, player contacts, and sport-specific technical actions, which are interspersed with periods of low to moderate intensity exercise that place significant physical demands on players (Mohr et al., 2003; Mohr et al., 2005; Rampinini et al., 2011; Akenhead et al., 2016; Beato and Drust, 2021). An inescapable consequence of these demands is fatigue, defined as a symptom of tiredness or weakness, underpinned by a complexity of physiological and psychological processes (Thomas et al., 2018). Therefore, when implementing recovery and regeneration protocols, it is of particular importance to practitioners working in professional soccer to identify whether fatigue originated from central [progressive, exercised-induced reduction in the voluntary activation (VA) of the muscle] and/or peripheral mechanisms (loss of muscle force caused by disturbances in sites at or distal to the neuromuscular junction; Gandevia, 2001). Markers of exercise-induced muscle damage (EIMD) such as maximum force production, plasma creatine kinease, and perceived muscle soreness, alongside decrements of physical performance reflective of neuromuscular function such as jump and sprint performance, have been identified as a consequence of participation in simulated and actual soccer match-play (Ispirlidis et al., 2008; Rampinini et al., 2011; Nedelec et al., 2012; Nedelec et al., 2014; Goodall et al., 2017). Altered neuromuscular function, decreased maximum voluntary force, perceptual fatigue, and muscle soreness caused from match-play can last for several days and not be resolved by 72 h post-exercise (Rampinini et al., 2011; Brownstein et al., 2017; Thomas et al., 2017).

While fatigue and the associated measures of function in response to match-play are relatively well-studied, the impact of regular soccer training has not been. This is despite the potential for training to elicit decrements in physical function, and even compound the fatigue experienced as a consequence of match-play. Previous studies have lacked ecological validity as typically a period of complete rest is prescribed after the match stimulus, whereas professional soccer players are exposed to further physical training in the days post-match (Rampinini et al., 2011; Brownstein et al., 2017; Thomas et al., 2017).

Concurrent training is the training arrangement when athletes combine maximal strength and endurance training within the same training cycle (Fyfe et al., 2014). Elite soccer players require a diverse range of fitness components, hence organising an appropriate training arrangement is a complex challenge (Bangsbo et al., 2006). Soccer teams routinely follow a concurrent training programme utilising maximal strength and explosive power exercises, alongside sport-specific conditioning games (Little and Williams, 2006). Research has shown the organisation of concurrent training, and recovery time between training bouts might be able to modulate changes in physical performance (Enright et al., 2015). High volumes of concurrent training are routinely prescribed throughout the training week, each with the potential to elicit decrements in neuromuscular function (Thomas et al., 2018). Indeed, in one recent study, professional soccer players completed their usual concurrent training programme of one pitch session, an upper-body strength session, and one full-body strength session in the 72 h following a competitive game, which resulted in impairments in isometric hamstring strength, CMJ height, and sprint speed, alongside an increase in muscle soreness (Nedelec et al., 2014). Furthermore, differing training methods such as heavy-resistance strength exercise, jumping exercise, and maximal sprinting were found to elicit decrements in muscle function that persist for 48 h post-intervention, alongside increased fatigue perceptions (Thomas et al., 2018). Further study is necessary to assess neuromuscular alteration in response to concurrent training methods commonly applied to the physical preparation of elite soccer players.

In support, a recent study assessed weekly neuromuscular performance capabilities in early-career professional soccer players and found significant fluctuations in isometric knee extensor strength, and evoked twitch force, across a 5-week in-season mesocycle (Clancy et al., 2022). Furthermore, in a similar cohort of youth academy soccer players, Fitzpatrick et al. (2019) demonstrated consistent reductions in jumping performance after a typical soccer training session, which might indicate a decrement in neuromuscular function. Neither study, however, investigated daily neuromuscular performance responses across a training microcycle. Quantifying the cumulative physical and perceptual consequences of a concurrent training week in academy soccer players would provide valuable information for science and medicine teams to manage training load effectively.

A major challenge to understanding the consequences of concurrent training in soccer is selecting appropriate assessment and monitoring tools to capture the complex underpinning to fatigue, which is likely multi-factorial. Practitioners and researchers have used several tools that include internal, external, and athlete self-reported measures, which might be important factors to make evidence-based decisions on appropriate training (Taylor et al., 2012; Buchheit et al., 2013a; Buchheit et al., 2013b; Akenhead and Nassis, 2016; Thorpe et al., 2016; Carling et al., 2018; Fitzpatrick et al., 2019). The interaction of numerous training load metrics, physiological responses, physical function measures, and performance indices has previously been investigated to identify measures and interactions of performance, fatigue and recovery (Wallace and Baumeister, 2002; Coutts et al., 2007; Elloumi et al., 2012; Mooney et al., 2013; Brink et al., 2018; Evans et al., 2022). However, no collective use of these measures has been examined, nor their incorporation into a suite of laboratory-standard testing to identify altered neuromuscular function to provide greater insight into the determinants of fatigue in professional academy players. Given the complexity of stressors in elite football, identifying a range of subjective and objective tools to act as valid, sensitive measures of fatigue and recovery might provide an improved understanding of the impact of training and inform on recovery, player readiness, and training efficacy.

The overarching aim was to understand the influence of professional soccer training on neuromuscular and cognitive function, perceptual mood, and wellness states in an elite academy cohort. Specifically, we aimed to 1) profile the etiology and recovery time-course of neuromuscular function in response to a mixed-content, standard training week; 2) concurrently examine physical performance, cognitive function, and perceptual measures of mood and wellness; and 3) assess a range of simple tests applied practitioners could use in the field to act as surrogate measures of neuromuscular function. We hypothesised that the acute effects of an initial strenuous day and the combined effects of 2 days training would show alterations in neuromuscular function, both central and peripheral in origin, which would persist for several days, with concurrent decrements in physical performance and perceptual wellness.

## Materials and Methods

### Participants

Ethical approval was granted by Northumbria University Faculty of Health and Life Sciences Ethics Committee, in accordance with the ethical standards established in the Declaration of Helsinki. Sixteen male academy scholarship Association Football players (four defenders, five midfielders, and seven forwards; age: 17 ± 1 year; stature: 1.77 ± 0.08 m; body mass: 66.1 ± 11.0 kg) from the U18 Professional Development League Two (South) gave written, informed consent to participate in the study. The cohort represents a convenience sample and hence overall numbers are limited by the size of the squad. All participants were free from injury for the preceding 3 months. The study was conducted in mid-season, where a typical week consisted of four pitch sessions, one competitive match, and two strength sessions. Participants reported to the training ground between 07:30 and 08:30 in a fed and hydrated state after consuming a standard mixed carbohydrate and protein breakfast and having refrained from consuming caffeine in the 12 h prior to each visit.

### Design

**FIGURE 1:**
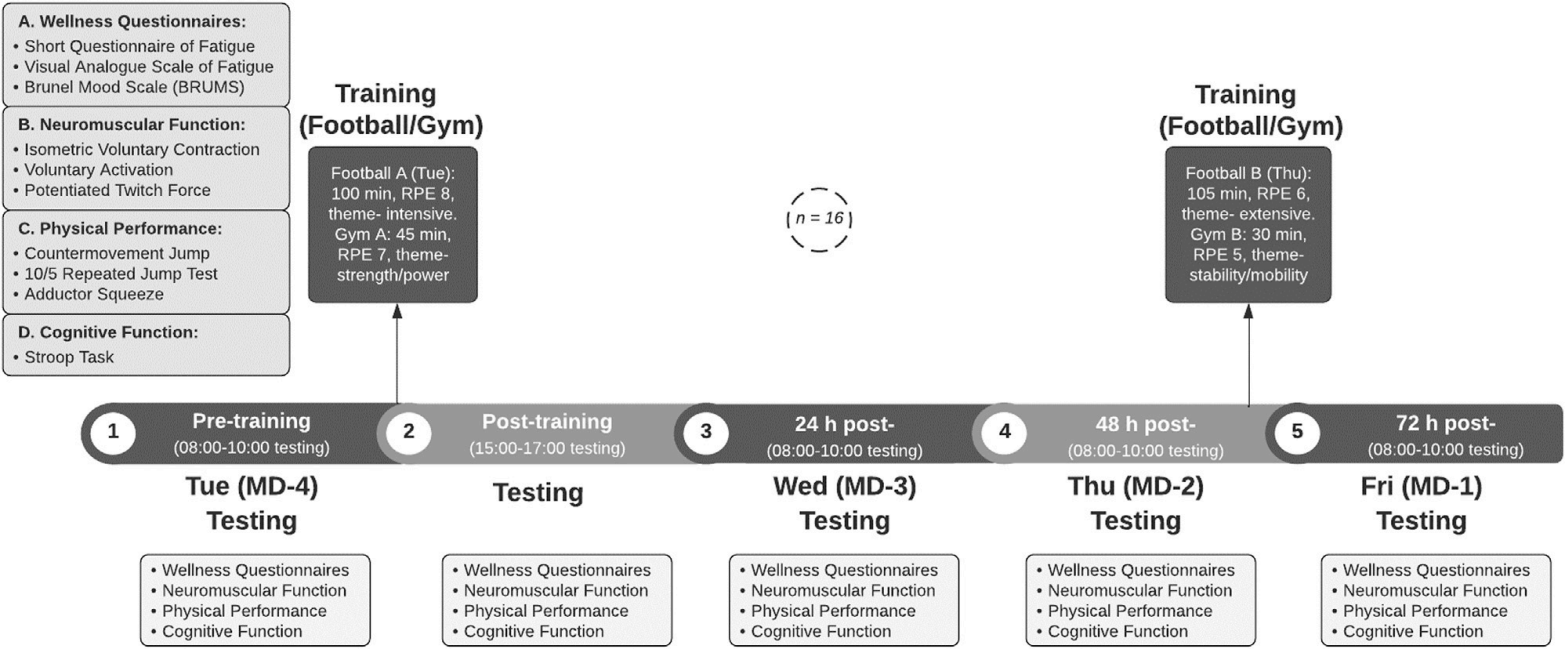
Schematic of experimental testing and training protocol.

Each participant completed one familiarisation session in the preceding week that contained all testing protocols. In the following week, five experimental sessions [pre- and post-strenuous session on day one (Tuesday, MD-4), and on subsequent days at 24, 48, and 72 h post-exercise] were conducted. The testing was completed at the mid-point of the U18 Professional Development League 2020/2021 season (early December 2020). A standard training week was chosen to carry out the experimental testing, with the U18 squad engaged in full-time training 4 days per week along with one competitive match per week (90 min duration). A competitive League game [90 min duration, average squad RPE (rating of perceived exertion) of 9 out of a maximum score of 10] was played on the preceding Saturday morning, followed by a day of rest away from the club on Sunday. On Monday (MD + 2), the training prior to the first experimental trial was a standard MD + 2 training session (75 min duration, average RPE 5) and hence served to ensure athletes reported for the first testing session Tuesday morning in the freshest state possible. As part of the standard training week, Tuesday (MD-4) represented the highest training load day of the week, followed by a rest day Wednesday (MD-3); a moderate training session on Thursday (MD-2); and a light match-preparation session on Friday (MD-1). Throughout the study, the players carried out their typical scholarship duties of training, educational lessons and player development sessions. The schematic of the experimental testing and training protocol is presented in Figure 1.

### Experimental Trial Outcome Measures

A range of neuromuscular, physical, perceptual, mood, and cognitive function tests were assessed throughout the training week. Testing protocols were administered between 30 and 120 min before the pitch training session began, with the post-session assessment taking place between 10 and 60 min following the gym session on experimental day one. The testing protocols were assessed at the same time on each subsequent day. All players performed a 5 min warm up on an indoor stationary bike before any tests were completed. The experimental protocol is presented in Figure 1.

## Procedures

### Training Physical Performance and Intensity

External [Global Positioning System (GPS), STATSports, Newry, Northern Ireland] and internal [session RPE; CR10-scale (Borg, 1985; Foster et al., 2001; Impellizzeri et al., 2004)] training loads were tracked across a standard training week. The pitch and gym-based training session durations and intensity were recorded. The portable GPS unit (Apex, STATSports, Newry, Northern Ireland) sampled at 10 Hz provided information on position and time, and a 952 Hz accelerometer measured accelerations. The validity and reliability of these units to estimate distance, velocity, immediate, and continuous movements during linear and multidirectional team sport movement activities have previously been shown (Coutts and Duffield, 2010; Varley et al., 2012; Beato et al., 2018; Thornton et al., 2019). The GPS units were placed inside a custom-made manufacturer-provided vest that kept the unit tightly pressed between the athlete’s scapulae and was turned on 30 min before commencement of training so that an adequate signal connection was made between the GPS receiver device and multiple satellites in the surrounding atmosphere. GPS and accelerometer-derived locomotor outputs that are typically used in professional football clubs were measured, including total distance (TD), high-speed running (HSR), distance covered at running velocities higher than 5.5 m s^−1^, total accelerations (>1 m s^−1^), total decelerations (>1 m s^−2^), sprint distance (distance covered at running velocities higher than 7.0 m s^−1^), number of sprints (number of times player was exposed to velocity above 7.0 m s^−1^) and maximum speed attained in session (Akenhead and Nassis, 2016).

### Perceptual Responses

Participants completed three psychometric testing questionnaires to measure perceived fatigue, readiness and mood. Participants completed the readiness to train and mood questionnaires on their mobile devices and the visual analogue scale questionnaire with paper and pen at the testing centre.

Participants completed an adapted version of the Short Questionnaire of Fatigue (Chatard et al., 2003; Elloumi et al., 2012), with the use of the Premier League’s Performance Management Application. This tool measured performance readiness *via* eight subjective measures of preparedness to train, namely-training difficulty, muscle soreness, nutritional quality, sleep, illness status, mood, mental status, and motivation. Each question was rated on a 5-point Likert Scale of −2 to +2, with verbal descriptors from “significantly harder/worse than usual” to “significantly lighter/better than usual”. Participants then completed the Brunel Mood Scale (BRUMS) questionnaire (Terry et al., 2003); a 24-item scale consisting of six mood factors (anger, depression, tension, vigour, fatigue, and confusion).

The players completed a further customised perceived wellness questionnaire [Visual Analogue Scale to Evaluate Fatigue Severity (VAS-F)] consisting of five subjective measures of fatigue, soreness, energy levels, levels of sleepiness, and readiness to train (Lee et al., 1991). Participants indicated on the 100 mm scale to the questions of “indicate how you are feeling right now?” For each measure, the question was stated such as “how fatigued do you feel?” and “how motivated to train do you feel?” Each scale was anchored with verbal descriptors “not at all” to “extremely.”

### Assessment of Neuromuscular Function

The maximum voluntary strength of the quadriceps and evoked responses *via* electrical stimulation of the femoral nerve were used to assess the contribution of central and peripheral adjustments in neuromuscular function and their subsequent recovery. Maximal voluntary force (MVC; N) was recorded from a calibrated load cell (Digitimer Strain Gauge 200 kg, LCM Systems, Newport, UK) during an isometric MVC of the knee extensor muscles. Participants sat in a custom-build chair with their hips and knees flexed at 90°. A load-cell was fixed to the chair, and subsequently attached to a non-compliant cuff which was positioned around the participant’s right ankle, superior to the ankle malleoli. Participants completed two warm up contractions at ∼50%–75% of perceived maximum, followed by three measured maximal isometric MVC repetitions (3–5 s duration), separated by 1–2 min rest (Brownstein et al., 2017). Motor nerve stimulation of the right femoral nerve was delivered at the highest point of each MVC manoeuvre and a single twitch was delivered 3 s post-contraction to measure potentiated twitch force (Q_tw,pot_) to allow the calculation of voluntary activation (VA; Merton, 1954). Single, electrical stimuli (200 μs pulse width) were administered using a constant-current stimulator (DS7AH, Digitimer Ltd., Hertfordshire, UK) with the cathode placed over the skin in the femoral triangle and the anode (both CF3200 Electrodes, Axelgaard Ltd., CA, United States) placed midway between the greater trochanter and the iliac crest (Thomas et al., 2017). To ensure consistent placement during the assessment days, the positions of the stimulating electrodes were marked with indelible ink. Electrical stimuli were first administered at rest in 20 mA stepwise increments from 20 mA until the maximum quadriceps twitch was produced to determine motor nerve threshold. Stimulation intensity was set at 130% to ensure supramaximal stimulus (203 ± 31 mA). There was a 1 min rest time interval between identification of stimulation intensity and the subsequent MVC.

Signals were amplified: gain ×300 for force (CED 1902; Cambridge Electronic Designs, Cambridge, UK), digitised (4 kHz; CED 1401, Cambridge Electronic Designs, Cambridge, UK) and analysed offline (v10 Spike2, CED). VA was assessed through the interpolated twitch technique (Merton, 1954) and quantified by comparing the amplitude of the superimposed twitch force (SIT) to the potentiated twitch (Q_tw,pot_) that was delivered at rest, 3 s after the MVC. The following equation was used offline:

Motor nerve VA (%)= [1 – (SIT/(Qtw,po))× 100]

### Assessment of Physical Function

Proceeding the MVC test, all participants performed a warm up of 10 body weight squats, followed by a 2-min rest period before completing two warm up maximal countermovement jumps (CMJ), followed by three maximal CMJ trials interspersed with 1-min recovery between repetitions. During each CMJ, participants were advised to stand in a typical ready position, with hands on their hips, feet shoulder-width apart and facing forwards. All jumps were strictly vertical using the optical timing system jump area (Optojump, Microgate, Italy) with participants instructed to perform the CMJ “as they normally would” using a self-selected squat depth and “to jump as high as they can.” Jump height (cm) was analysed off-line, where the trial with the maximal score was recorded.

Following a 2-min rest period, the 10 to 5 repeated jump test (10/5 RJT) (Harper, 2011) was performed using the Optojump to measure the participants’ reactive strength index (RSI). Participants were instructed to keep their hands on their hips, and verbal instructions were given to “jump as high as possible” and “try and minimise your contact time on the ground” for a series of 10 maximal continuous jumps, of which the mean of the five best RSI scores were taken for data analysis. RSI was calculated as the ratio between jump height (m) and ground contact time (s). To ensure the 10/5 RJT was assessing a sufficiently fast stretch-shortening cycle, a maximum ground contact time of 200 ms was allowed during each jump; contact times exceeding this limit were discarded. The participants completed two sets, with 2 min rest between. The mean of the 5 best RSI (jump height/ground contact time) was calculated off-line and used for data analysis.

Participants performed a bilateral isometric contraction of the adductor muscles using a pressure cuff from a sphygmomanometer (FlexiPort Blood Pressure Cuff, Welch Allyn, Skaneateles, NY) pre-inflated to 10 mmHg which have previously shown good intrarater reliability [0° position intraclass correlation coefficient (ICC) = 0.89, 45° position ICC = 0.92, Delahunt et al., 2011]. Participants lay supine in two test positions [0° (legs flat) and 45° of hip flexion] assessed by a goniometer. Trials were completed with participants’ back, hips, and feet firmly on the ground. The cuff was placed between the participant’s knees with the instruction to squeeze the cuff as hard as possible. The highest-pressure value displayed in mmHg on the sphygmomanometer dial was recorded during each maximal adductor squeeze test. Players completed two maximum efforts at each angle with 30 s recovery between repetitions (Delahunt et al., 2011). The trial with the maximal score was recorded.

### Assessment of Cognitive Function

The Stroop task was used to assess cognitive executive function such as inhibition of stimuli, selective attention, and assessment of complex tasks (Wallace and Baumeister, 2002). Participants sat in a quiet room with no distraction to complete the cognitive reactive performance assessment. Cognitive measures were administrated using the Computerised Mental Performance Assessment System (COMPASS, Northumbria University, UK). The test was scored for both congruent and incongruent correct response, reaction time (ms) and accuracy (%) in response to 30 stimuli. Two trials of 30 items were carried out, with 2 min rest between trials.

### Statistical Analyses

Data are presented as mean ± SD. All data were screened for outliers using boxplots and normality of distribution was assessed using the Shapiro-Wilks test. One-way repeated measures ANOVA was used to assess changes in each outcome measure over time (pre-, post-, 24, 48, and 72 h). Assumptions of sphericity were assessed by Mauchly’s test, and where necessary variables were adjusted using the Greenhouse-Geisser correction. Bonferroni post-hoc tests were performed for pairwise comparisons. Where data were not normally distributed, and for rating scale questionnaire variables, Friedman’s test with pairwise comparisons were carried out. Significance was accepted at ≤0.05. To assess the magnitude of change, 95% confidence intervals (CI) were determined. To determine the size of the change, Cohen’s effect size (ES; Cohen, 1988) were calculated for pairwise comparisons using Microsoft Excel (2016). Following the ranges set by Cohen (1988), ES were interpreted as trivial (<0.2), small (0.2–0.50), moderate (0.5–0.8), or large (>0.8). Pearson product-moment correlation coefficients were used to assess the relationship between pre-training to 24 h post changes of neuromuscular function, physical performance and perceptual responses. All data were analysed using Statistical Package for Social Sciences (SPSS version 26.0) (Chicago, Illinois, United States).

## Results

### Training Physical Performance and Intensity

Physical activity outputs and internal measures of load for both football training sessions (Table 1) and resistance training sessions (Table 2) are presented below.

**TABLE 1:** Football training sessions one and two.

Football session A (Tue)	TD (m)	HSR (m)	ACCEL (#)	DECEL (#)	SD (m)
Absolute	7,662	214	130	116	17
Per minute	76.6	2.1	1.3	1.2	0.17
^a^Relative %	69%	59%	149%	122%	51%
Football session B (Thu)					
Absolute	7,089	141	68	57	6
Per minute	67.5	1.3	0.7	0.6	0.06
Relative %	60%	37%	74%	57%	18%

100 min, RPE 8 (±0.6), intensive theme. Content—speed warm up (12 min), technical practice (15 min), passing and receiving (10 min), transfer possession practice (19 min), possession practice (14 min), phase of play (15 min), conditioning runs (3 × 3 min tabata protocol). 105 min, RPE 6 (± 0.7), extensive theme. Content—physical warm up (6 min), passing and receiving (8 min), 11v11 phase of play (26 min), set-piece practice (20 min), 11v11 large-sided game (36 min). TD, total distance; HSR, high-speed running, distance covered at running velocities higher than 5.5 m s; ACCEL, accelerations, >1 m s^−2^; DECEL, decelerations, >1 m s^−2^; SD, sprint distance, distance covered at running velocities higher than 7.0 m s.

a Relative %, training variable per minute compared to typical match variable per minute.

**TABLE 2:** Resistance session A (Tue) and session B (Thu).

Resistance session A (Tue)	
Exercise	(sets | repetitions | % 1RM)
Single leg box jump	4 | 4 | Body mass
Dumbell squat jump	3 | 4 | 10%–20% 1RM
Barbell back squat	3 | 8 | 60%–70% 1RM
Nordics	3 | 4 | Body mass
Loaded lateral lunge	3 | 8 | 60%–70% 1RM
Dumbell bench press	3 | 6 | 70%–80% 1RM
Dumbell bent over row	3 | 6 | 80%–85% 1RM
Resistance session B (Thu)	
Squat	3 | 12 | Body mass
Split squat	3 | 16 | Body mass
Isometric adductor bridge	3 | 30 s | Body mass
Isometric hamstring bridge	3 | 30 s | Body mass
Standing calf raises	3 | 30 | Body mass
Press up	2 | 20 | Body mass
Deadbug	2 | 20 | Body mass
Frontal plank	2 | 60 s | Body mass

45 min, RPE 7 (±0.6), strength and power theme. 30 min, RPE 5 (±0.4), stability and mobility theme.

### Perceptual Responses

Perceptual responses from the Visual Analogue Scale (VAS) to Evaluate Fatigue Severity, the Short Questionnaire of Fatigue (SQF) Likert scale, and the Brunel Mood Scale (BRUMS) are shown in Table 3. VAS fatigue showed changes over time [F_(2)_ = 17.5; *p* < 0.001], alongside increases in muscle soreness [F_(4)_ = 21.5; *p* < 0.001], levels of sleepiness [F_(4)_ = 5.2; *p* = 0.001], and a decrease in energy levels [F_(4)_ = 8.3; *p* < 0.001] and readiness to train [F_(2)_ = 11.4; *p* < 0.001]. Perceptions of fatigue, muscle soreness, and sleepiness increased post-training, and had recovered by 24 h (sleepiness) and 48 h (fatigue; muscle soreness). Furthermore, perceived levels of energy and readiness to train were decreased at post-training, before recovering at 24 h (energy levels) and 48 h (readiness to train).

**TABLE 3:** Perceptual responses at pre-, post-, and 24, 48, and 72 h post-training day (*n* = 16).

	Pre-	Post-	24 h	48 h	72 h
Perceptual responses measured *via* visual analogue scale (mm)					
Fatigue	43 ± 18	72 ± 16*	69 ± 14*	49 ± 16	47 ± 15
Soreness	39 ± 20	68 ± 18*	69 ± 15*	53 ± 15	43 ± 15
Sleepiness	40 ± 19	55 ± 13*	51 ± 21	43 ± 14	38 ± 15
Energy	51 ± 11	31 ± 16*	36 ± 18	48 ± 13	48 ± 13
Readiness to train	57 ± 13	33 ± 19*	37 ± 16*	52 ± 16	57 ± 13
Perceptual responses measured *via* Likert scale (−2 to +2)					
Training difficulty	0.25 ± 0.58	−1.19 ± 1.05*	−1.19 ± 0.91*	−0.81 ± 1.05	−0.06 ± 0.44
Nutrition	0.19 ± 0.54	0.00 ± 0.37	0.13 ± 0.34	0.06 ± 0.057	0.00 ± 0.00
Quality of sleep	0.19 ± 0.75	0.00 ± 0.52	−0.25 ± 0.58	−0.13 ± 0.72	0.19 ± 0.54
Muscle soreness	−0.38 ± 0.50	−1.38 ± 0.62*	−1.19 ± 0.66*	−0.81 ± 0.66	−0.56 ± 0.56
Illness	0.06 ± 0.25	0.06 ± 0.25	0.00 ± 0.37	−0.06 ± 0.25	0.00 ± 0.00
Mental state	0.19 ± 0.40	−0.13 ± 0.50	−0.06 ± 0.25	0.00 ± 0.00	0.06 ± 0.44
Stress levels	0.00 ± 0.00	−0.13 ± 0.50	−0.06 ± 0.25	0.00 ± 0.00	−0.06 ± 0.25
Motivation	0.38 ± 0.62	0.13 ± 0.72	−0.06 ± 0.57	0.06 ± 0.25	0.19 ± 0.40
Perceptual responses measured *via* BRUMS subscales (0–4)					
Tension	0.38 ± 0.89	0.31 ± 0.70	0.13 ± 0.50	0.13 ± 0.34	0.56 ± 1.03
Depression	0.13 ± 0.34	1.06 ± 2.38	0.25 ± 0.77	0.19 ± 0.40	0.06 ± 0.25
Anger	0.31 ± 0.87	0.88 ± 1.89	0.19 ± 0.54	0.25 ± 0.68	0.31 ± 0.79
Vigour	3.06 ± 3.30	2.63 ± 3.38	1.25 ± 2.11	2.19 ± 2.54	2.25 ± 3.13
Fatigue	1.94 ± 1.81	8.38 ± 4.08*	4.13 ± 3.54	2.94 ± 2.57	2.25 ± 2.52
Confused	0.13 ± 0.50	0.19 ± 0.75	0.19 ± 0.75	0.19 ± 0.54	0.25 ± 0.77

Values are mean ± SD. Significant differences in comparison with baseline indicated by *p < 0.05.

For the SQF, Friedman Tests were administrated as assumptions of normality were violated for all questions. Training difficulty of the previous session showed a time effect (χ^2^(4) = 26.9, *p* < 0.001), alongside an increase in muscle soreness (χ^2^(4) = 34.2, *p* < 0.001). Perceived training difficulty and muscle soreness were increased post-training and recovered by 48 h post-exercise.

In the BRUMS, an increase in levels of fatigue across time was observed [F_(2)_ = 19.0; *p* < 0.001]. Pairwise comparisons showed higher perceptual fatigue than pre-training at post- (*p* < 0.001), and fatigue was higher at post-training than at 24 h (*p* = 0.023), 48 h (*p* = 0.001), and 72 h (*p* = 0.001). Full details of pairwise comparisons for all perceptual measures are shown in Table 3.

### Neuromuscular Function

**FIGURE 2:**
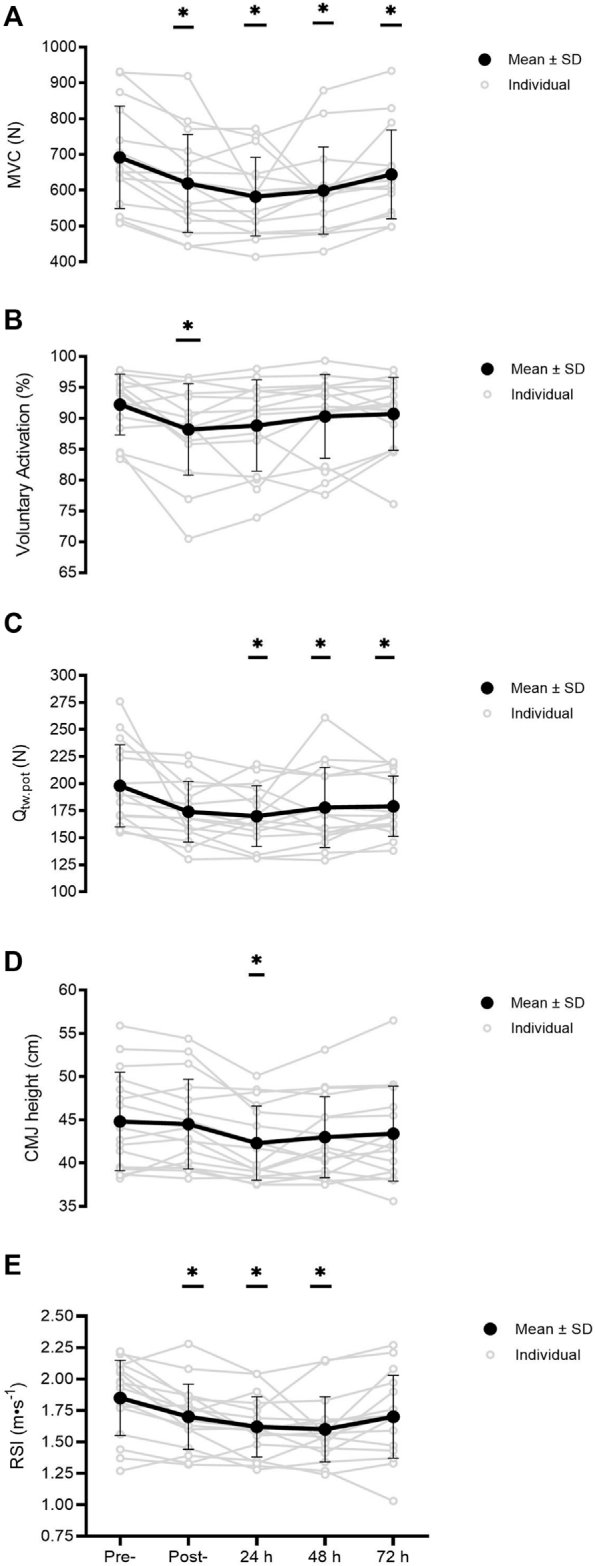
MVC, maximal voluntary force **(A)** VA, voluntary activation **(B)** Q_tw.pot_, quadriceps potentiated twitch force **(C)** CMJ, countermovement jump height **(D)** and RSI, reactive strength index **(E)** measured pre-, post-, and at 24, 48, and 72 h post-training (*n* = 16). Significant differences in comparison with baseline indicated by *p < 0.05. Individual responses are plotted with grey circles and lines, with black dots and connecting lines representing the mean scores, and error bars representing the ± SD.

Changes in MVC showed a time effect [F_(2)_ = 10.0; *p* < 0.001, Figure 2A]. *Post hoc* analysis showed MVC force was reduced by 11 ± 7% from pre-to post-training (692 ± 143 vs. 619 ± 137 N, *p* = 0.001, 95% CI 289-117 N, *d* = 0.52), remained depressed at 24 h by 15 ± 8% (582 ± 110 N, *p* < 0.001, CI 47–173 N, *d* = 0.86) and 48 h by 13 ± 11% (599 ± 122 N, *p* = 0.018, CI 12–173 N, *d* = 0.70), and still had not recovered by 72 h at −6 ± 6% (644 ± 124 N, *p* = 0.032, CI 3–93 N, *d* = 0.36) compared to pre-training levels. VA also showed a time effect [F_(4)_ = 4.3; *p* = 0.004, Figure 2B] and was only reduced at the post-trial time point by 4 ± 5% from baseline (92 ± 5 vs. 88 ± 7%, *p* = 0.038, 95% CI 0.2%–8%, *d* = 0.63), but recovered by 24 h (*p* = 0.087). A change in Q_tw,pot_ across time was observed [F_(4)_ = 6.2; *p* < 0.001, Figure 2C]. *Post hoc* analysis showed Q_tw,pot_ was maintained post-session with a non-significant decrease of −11 ± 12% (174 ± 28 N, *p* = 0.123, CI −4–52 N, *d* = 0.73), and was reduced at 24 h post training by 13 ± 11% (198 ± 38 vs. 170 ± 28 N, *p* = 0.013, CI 5–51 N, *d* = 0.84), remained depressed at 48 h by 10 ± 10% (178 ± 37 N, *p* = 0.035, CI 1–40 N, *d* = 0.55), and had not recovered by 72 h at −9 ± 9% (179 ± 28 N, *p* = 0.025, CI 2–37 N, *d* = 0.58).

### Physical Function

A change in CMJ height was observed across time [F_(4)_ = 6.3; *p* < 0.001, Figure 2D]. Jump height was maintained post-session with a non-significant drop of −0.4 ± 4% (44.5 ± 5.7 cm, *p* = 1.000, CI −1.0–1.6 cm, *d* = 0.05), though jump height decreased from baseline levels at 24 h by 5 ± 5% (44.8 ± 5.7 vs. 42.3 ± 4.3 cm, *p* = 0.009, CI 0.5–4.5 cm, *d* = 0.50), and recovered by 48 h (*p* = 0.265). RSI derived through the 10/5 RJT showed a time effect [F_(4)_ = 9.6; *p* < 0.001, Figure 2E). Pairwise comparisons showed RSI was reduced by 8 ± 9% from pre- to post-training (1.85 ± 0.30 vs. 1.70 ± 0.26 AU, *p* = 0.027, 95% CI 0.01–0.30 AU, *d* = 0.54), was depressed at 24 h by 12 ± 10% (1.62 ± 0.24 AU, *p* = 0.003, CI 0.07–0.40 AU, *d* = 0.85), remained depressed at 48 h by 13 ± 11% (1.60 ± 0.26 AU, *p* = 0.004, CI 0.07–0.45 AU, *d* = 0.91), but recovered by 72 h (*p* = 0.059). No significant changes were found for the adductor squeeze test at both 0° hip angle (*p* = 0.192) and 45° hip angle of the participants (*p* = 0.341).

### Cognitive Function

There was no effect across time in any cognitive function results from the Stroop task (pre-, post-, 24, 48, and 72 h). All reaction time values showed no change (*p* > 0.05 for overall reaction time, correct reaction time, congruent reaction time, incongruent reaction time, congruent correct answers reaction time, and incongruent correct answers reaction time). The Stroop task’s measures of correct answer percentages all violated the assumption of normality and a Friedman Test was administered. There were no statistical changes over time (*p* > 0.05) for percentage correct answers, percentage correct congruent answers, and percentage correct incongruent answers at any time point.

### Relationship Between Neuromuscular Variables and Physical and Perceptual Measures

The relationships between the changes at 24 h post-training of neuromuscular function variables and selected physical function and perceptual responses are reported in Table 4. A large association was shown between reductions in motor nerve VA and MVC (*r* = 0.609, *p* < 0.05) and countermovement jump performance decrements at 24 h (*r* = 0.601, *p* < 0.05). While reductions in potentiated twitch force showed large associations with visual analogue scale perceptual measures fatigue (*r* = 0.539, *p* < 0.05) and soreness (*r* = 0.533, *p* < 0.05), no other significant relationships were identified at the 24 h post-training time-point between neuromuscular function variables and other physical function or perceived measures (*p* > 0.05, Table 4).

**TABLE 4:** Pearson product-moment correlation coefficients between neuromuscular variables and physical function and perceptual measures (% changes from pre-to 24 h post-exercise).

	Q_tw,pot_		Motor nerve VA	
	*r*	*p*	*r*	*p*
MVC	0.440	0.100	0.609*	0.016
CMJ	−0.293	0.289	0.601*	0.018
10/5 RJT-RSI	0.072	0.798	0.303	0.271
VAS-fatigue	0.539*	0.038	0.012	0.967
VAS-RtT	−0.011	0.969	−0.259	0.351
VAS-soreness	0.533*	0.041	−0.085	0.763

Significant correlation indicated by *p < 0.05. Q_tw,pot_, quadriceps potentiated twitch force; VA, voluntary activation; CMJ, countermovement jump; 10/5 RJT-RSI, 10 to 5 repeated jump test-reactive strength index; VAS-fatigue, visual analogue scale fatigue question; VAS-RtT, visual analogue scale readiness to train question; VAS-soreness, visual analogue scale soreness question.

## Discussion

The primary aim of this study was to profile the etiology and recovery time-course of neuromuscular function in response to a mixed-content standard training week in elite youth soccer players. The training week elicited substantial impairments in neuromuscular function that were largely attributable to reductions in contractile function, which persisted for the 72 h measurement period across the training week. A secondary aim was to examine the effect the academy training programme had on typically used applied measures of physical performance, cognitive function, and perceptual mood and wellness states and whether simple surrogate measures could be applied by practitioners in the field to identify neuromuscular alterations. At successive time-points there was evidence of impairments in the players’ ability to generate maximum force (knee-extensor MVC) and RSI derived from the 10/5 RJT. However, perceptual measures of fatigue and countermovement jump performance decrements recovered by 48 h post-exercise, showing a divergence of findings when attempting to identify tiredness/weakness and surrogate measures of fatigue. Knee-extensor MVC and the 10/5 RJT showed similar decline and time-course of recovery as skeletal muscle function, and so might be appropriate tools to indirectly measure neuromuscular function peripheral processes. To attain a comprehensive picture of athlete status, practitioners are recommended to use multiple assessments (objective and subjective measures) due to a divergence in recovery of physical function and perceptual measures.

### Neuromuscular Adjustment Following Training

This is the first study to measure neuromuscular function across a typical training week of elite youth soccer players. The ability to generate maximal force (MVC) was reduced immediately post-session (−11%) and remained depressed up to 72 h post (−6%). MVC was impaired immediately post and, concurrent with reductions in potentiated twitch force, was still present at 72 h post-session. The prolonged reduction in MVC, which is widely used as an indicator of EIMD and a global measure of neuromuscular function (Goodall et al., 2017), added to the presence of perceived muscle soreness, indicates that the strenuous training and cumulative sessions in the present study elicited substantial muscle damage. These decrements had not fully recovered 24 h prior to the next competitive game which has important implications for professional support staff to ensure players approach competitive games in a fit and ready state to perform.

Declines in VA were substantial immediately post-session (−4%), but largely recovered by 24 h, whereas impairment of contractile function persisted for at least 72 h. The time-course recovery of VA contrasted with previous studies of football match-play that found a suppression until 48 h (Rampinini et al., 2011; Brownstein et al., 2017) and 72 h (Thomas et al., 2017). The slower time course of VA recovery in previous work (Thomas et al., 2017) could be explained by the contrasting timing in the season of the investigations (late off-season/early pre-season versus mid-season of the present study) when the players were perhaps less physically accustomed to the match-play demands. Alternatively, the divergent findings might be due to the differing stimuli of simulated and competitive match-play in contrast to the acute and cumulative effects of a standard training week. The acute neuromuscular responses immediately post-exercise (−11% for MVC, −4% for VA, −11% for Q_tw,pot_) were less than previous data in response to simulated/competitive match-play (decrease of 14%–20% for MVC, 8%–15% for VA, 14%–23% for Q_tw,pot_). This suggests that neuromuscular alteration was prominent post-session, though the magnitude of impairment was less than in previous observations. Furthermore, in previous studies, testing was carried out immediately post competitive/simulated match, whereas in the present study, with the inclusion of an afternoon gym strength session, there was a delay between the end of the pitch session and post-training day measurements. Taking into consideration the differing demands of the pitch and gym session, this might also explain the magnitude of decrements. Impairments in neuromuscular function, indicated by deficits in contractile function and/or the central nervous system’s ability to activate the muscle (Gandevia, 2001), dissipates swiftly after metabolically-demanding exercise that involves low mechanical stress (Skurvydas et al., 2016). It is hence plausible the alterations in neuromuscular function in soccer players is a consequence of muscular damage and the ensuing inflammatory response (Howatson and van Someren, 2008) caused by repeated eccentric contractions involved in the strenuous training sessions (Ascenao et al., 2008; Brownstein et al., 2017). The findings of the present study indicate neuromuscular function alterations from a central origin are immediate post-session but do not persist across the training week. Therefore, applied practitioners should not focus their match-prep and regeneration strategies on recovering central processes.

Conversely, impairment at skeletal muscle (reduction in Q_tw,pot_) was observed at 24 h (−13%) and was still present for at least 72 h (−9%). These results were in accordance with previous work that showed slower recovery of muscle contractility compared to central processes after soccer match-play and extend these results to a typical mixed-training stimulus in soccer (Thomas et al., 2017). This suggests the impairment in muscle function observed (the persistent reduction in MVC) was largely attributable to adjustments in skeletal muscle function. This contrasts with similar studies where potentiated twitch force returned to baseline by 24 h post-match-play (Rampinini et al., 2011; Brownstein et al., 2017). These studies, however, were in response to competitive and simulated matches, rather than the cumulative effects of a standard training week, so comparisons should be made with caution. Additionally, the populations (semi-pro adult vs. professional youth players) are not directly comparable as high intensity running distance is lower for semi-pro players than professional players (Mohr et al., 2003; Brownstein et al., 2017) and other physical qualities can differ between well-trained and lesser trained athletes (Birat et al., 2018). Moreover, physiological responses can differ between adults and those still developing, and growth and maturation can impact young players’ physiological development (Falk and Dotan., 2006; Bontemps et al., 2019; Eisenmann et al., 2020). Additionally, previous studies lacked ecological validity because complete rest was given as recovery, whereas the current cohort trained within 48 h of the initial training bout (a typical schedule in professional football). From a practitioner perspective, it highlights the need to ensure that peripheral (skeletal muscle) function should remain the primary target of recovery and regeneration in soccer players following intense exercise and match play (Thomas et al., 2017). It also highlights the need for skeletal muscle development and robustness in these athletes to participate and flourish in a full time professional programme. This supports the inclusion of an appropriate physical development programme and supplementary strength training practices to promote muscular adaptations to help youth players withstand the high demands of training (Clancy et al., 2022). When devising regeneration strategies to limit fatigue and promote recovery, it is important to understand the stressors that cause performance decrements and delayed recovery before implementing the appropriate intervention (Howatson et al., 2016). Thus, the results above have important implications for both the recovery modalities and long term athletic development programmes applied in professional soccer clubs.

### Recovery of Physical Function

CMJ and RSI from the 10/5 RJT showed impairments post-session (RSI), 24 h (CMJ and RSI) and at 48 h post-session (RSI). RSI decrements followed a similar timeline as MVC and contractile function decrements, with a reduction immediately post-training (−8%), remained depressed at 24 h (−12%), although was recovered by 72 h post-exercise. CMJ height was maintained immediately post-session but was impaired at 24 h (−5%). The prolonged reductions in RSI and faster recovery of CMJ was similar to the mixed-findings of previous literature (Ispirlidis et al., 2008; Magalhaes et al., 2010; Rampinini et al., 2011; Nedelec et al., 2012; Nedelec et al., 2014; Brownstein et al., 2017; Thomas et al., 2017). RSI and muscle contractile function (potentiated twitch force, Q_tw,pot_) showed similar temporal change and characteristics across the training week, giving the 10/5 RJT potential as a simple measure of muscular function for practitioners in the field.

Physical performance jump tests (CMJ, Squat Jump, Drop Jump) are often used in football settings to identify athlete status (Ispirlidis et al., 2008; Magalhaes et al., 2010; Thorpe et al., 2015; Akenhead and Nassis, 2016). In both the current study and previous work, a relationship between CMJ height and VA measures have been identified (Brownstein et al., 2017). However, vigilance is required in using CMJ height as a measure of neuromuscular fatigue; Thorpe et al. (2017) found a poor relationship between CMJ height and the daily fluctuations in training load and therefore might not be a sensitive measure of physical status (Thorpe et al., 2017). Critically however, football clubs typically carry out neuromuscular and perceptual assessments at 24 h post, and there was a decrease in CMJ and a strong relationship between CMJ height and central nervous system alterations at that time-point in the current study. CMJ might be worthy of inclusion in a comprehensive battery of monitoring protocols as a tool to measure physical function 24 h post-training.

In the present study, adductor squeeze scores showed no changes in response to training and is therefore an inappropriate measure to examine physical changes from soccer-specific training. Though a recent study of male footballers found groin injuries accounted for 18% of all time-loss injuries (Mosler et al., 2018), and a relationship was found between hip adductor strength and the incidence of groin injuries during two competitive seasons of professional football players (Moreno-Perez et al., 2019), the lack of effect across time in the present study implies the daily measurement of adductor squeeze scores might not be a worthwhile monitoring tool. This is an important point for applied practitioners in soccer, as clinicians often test adductor strength through the use of isometric squeeze tests to monitor fluctuations in scores as a risk factor in groin injury (Light and Thorborg, 2016). The results of the present study show, however, the limited use of adductor squeeze scores to understand fatigue and recovery.

### Recovery of Cognitive Function

The present study showed no changes in cognitive function, which contrasts with other well-trained athletes (rugby players), who exhibited decrements in cognitive function (choice reaction time and selective inhibition) in response to strenuous exercise (Browne et al., 2017; Costello et al., 2021). There is currently no consensus on the relationship between acute exercise and cognitive performance (Browne et al., 2017). Meta-analyses concluded high-intensity exercise had a small, positive effect on executive performance (Chang et al., 2012; Moreau and Chou, 2019), in contrast to a further meta-analysis which reported a small negative effect following exercise, though the effect of exercise on cognitive performance is dependent on the specific domain being assessed (Lambourne and Tomporowski, 2010). In accordance with the ambiguous results regarding complex cognitive processes, the findings of the present study emphasised the inability of the Stroop task to detect changes in stimuli inhibition, selective attention, cognitive flexibility and assessment of complex tasks in response to exercise in the present cohort. A possible explanation is the Stroop task was not an appropriate test to identify the nuanced and multi-factorial executive cognitive functions involved in team sports and so other options could be explored.

### Recovery of Perceptual Responses

Strenuous training resulted in increased fatigue, muscle soreness, training difficulty, and reduced readiness to train. Whereas neuromuscular alterations and reduced muscle function were present up to 72 h post-exercise, perceptual responses returned to baseline by 48 h after exercise. An explanation might be the psycho-emotional stress and expectations preceding the physical challenge might recover quicker than the inflammatory response that might cause decrements in muscular function (Kiely, 2018). Daily subjective scores of wellness have shown to correlate with daily fluctuations of training load, indicating a possible dose-response relationship (Thorpe et al., 2017). However, caution is advised in interpreting the results, and the use of athlete self-report measures as measures of fatigue and readiness in the applied world, as perceptual measures have shown to have poor test-retest reliability (Fitzpatrick et al., 2019). The lack of mood, stress or mental fatigue changes experienced contrasted with previous studies (Thompson et al., 2020), which further supports such measures being more suitable as status indicators and facilitators of communication only (Duignan et al., 2020).

### Training Physical Performance and Intensity

The external training loads, such as running distance, high speed running, and accelerations were similar to previously published work in similar cohorts (Fitzpatrick et al., 2019; West et al., 2021). Internal training load (sRPE; 600–800 AU) and training volume (duration; 70–105 min) for training sessions were similar to previously published data in elite English Premier League soccer players, and elite youth academy players, suggesting the physical demands of the strenuous training day and subsequent sessions across the standard week were comparable to high performing professional and elite junior soccer players (Wrigley et al., 2012; Malone et al., 2015a; Malone et al., 2015b). This, along with the consistent measurement methods and prolonged physical decrements exhibited, demonstrates the robust nature of the study and data.

### Limitations and Future Directions

Despite the ecological validity of the study, a potential limitation was that the demands of football training are inherently variable (Hill-Haas et al., 2008; Mateus et al., 2021) and hence the magnitude and time-course of recovery might show high inter-participant variability. Though not practically feasible in an applied setting, there was no control group as part of the study. Pre-training measures of neuromuscular function were not carried out on athletes in a fully-rested state, as the participants trained 24 h preceding testing. Additionally, the testing was in-season, so there might have been a cumulative effect of training over time, with the previous competitive game played 72 h previously (Saturday). However, the physical activity profile of the Monday training session was a standard MD + 2 academy training session for the players (average RPE 5), and the Saturday-Saturday games programme with five training sessions in between is a typical professional academy programme (Wrigley et al., 2012; Fitzpatrick et al., 2019; Hannon et al., 2021). Evoked twitch responses from supramaximal electrical stimulation of the femoral nerve can vary due to electrode placement, joint position, and issues affecting repeated testing procedures across multiple days. To ensure testing reliability, the participants were comprehensively familiarised with all procedures. To minimise variability, the positions of the stimulating electrodes were marked with indelible ink to ensure consistent placement.

## Conclusion

A typical mixed-content training week in youth professional soccer players elicits persistent decrements in neuromuscular function, which do not fully recover the day before the subsequent game. The substantial impairments in neuromuscular function were largely attributable to reductions in contractile function (peripheral mechanisms). These mechanical factors can influence the excitation-contraction coupling process negatively (Allen et al., 2008). This was likely the consequence of significant mechanical stress enacted on the muscle fibres during training, which can result in sarcolemma impairment, muscle fibre damge, and cellular Ca^2+^ interference (Skurvydas et al., 2011; Brownstein et al., 2017). The acute effects of a demanding day combined with the cumulative effects of two training days also showed physical performance decrements, increased perceptions of soreness and fatigue, and decreased readiness to train. To attain a holistic overview of athlete status, it is prudent to use both objective and subjective measures, due to a divergence in recovery of physical function and perceived fatigue (Saw et al., 2017). Hence, a combination of measures of neuromuscular function, readiness and recovery when monitoring the training response can provide a comprehensive picture of a player’s preparedness to train and perform. Collectively the findings can inform training, preparation, and recovery activities to maximise longer-term player development and athletic progression at this critical point in their maturation.

## Practical Applications

Training-related neuromuscular alterations and fatigue persists throughout the training week and is determined by a combination of central, peripheral and perceptual factors. Neuromuscular alterations are substantial and even at 72 h post there is still evidence of impaired function, which is the day before the next competitive match. Professional support staff should be aware of the acute and cumulative effects of training and must be particularly cognisant of ensuring these youth players approach competitive games in a fit and ready state. How the training programme is organised and the physical preparation prescribed will depend on each academy’s philosophy regarding their desire and necessity to win competitive youth games versus their ethos and approach to long-term athletic and player development.

Practitioners should focus on the recovery and restitution of muscle damage and mechanical stress in the days post-strenuous training in preparation for performance. Utilising recovery methods that focus on the resolution of muscle function and soreness, such as cold water immersion, compression garments, and the application of garments fitted with cooled phase change material is advised, (Leeder et al., 2012; Hill et al., 2014; Clifford et al., 2018; Kwiecien et al., 2018; McHugh et al., 2018). Though further investigation is needed on the efficacy of various modalities in elite athletes (Barnett, 2006). It is further proposed to utilise periodizing strategies to consolidate recovery throughout a training period but also to enhance adaptation (Thorpe, 2021). When prescribing the appropriate recovery modality, practitioners must take into account the period of the season and the mechanical/metabolic/cognitive demand of the training stimulus applied (Kellmann et al., 2018). However, getting the basic principles correct of training, nutrition, hydration, sleep hygiene, and appropriate rest between exercise must remain the priority (Howatson et al., 2016).

Furthermore, it highlights the need for youth players to follow an appropriate physical development programme incorporating highly demanding field-based sport-specific movements such as accelerations, changes of direction, eccentric movements, and sprints. The programme should also include supplementary strength training and skeletal muscle development to promote physical robustness to withstand training-induced muscle function impairments in preparation for the demands of high intensity soccer training. The similar decline and time-course of recovery of RSI and contractile function indicate the 10/5 RJT might be an appropriate tool for practitioners to use in the applied field as a surrogate measure of peripheral neuromuscular alteration.

## Data Availability

The raw data supporting the conclusion of this article will be made available by the authors, without undue reservation.
